# Percutaneous, Imaging-Guided Biopsy of Bone Metastases

**DOI:** 10.3390/diagnostics8020025

**Published:** 2018-04-18

**Authors:** Dimitrios Filippiadis, Argyro Mazioti, Alexios Kelekis

**Affiliations:** 2nd Radiology Department, University General Hospital “ATTIKON”, Medical School, National and Kapodistrian University of Athens, 12462 Athens, Greece; argyromazioti@yahoo.gr (A.M.); akelekis@med.uoa.gr (A.K.)

**Keywords:** bone, metastasis, percutaneous, biopsy, imaging-guided

## Abstract

Approximately 70% of cancer patients will eventually develop bone metastases. Spine, due to the abundance of red marrow in the vertebral bodies and the communication of deep thoracic-pelvic veins with valve-less vertebral venous plexuses, is the most common site of osseous metastatic disease. Open biopsies run the risk of destabilizing an already diseased spinal or peripheral skeleton segment. Percutaneous biopsies obviate such issues and provide immediate confirmation of correct needle location in the area of interest. Indications for percutaneous bone biopsy include lesion characterization, optimal treatment and tumor recurrence identification, as well as tumor response and recurrence rate prediction. Predicting recurrence in curative cases could help in treatment stratification, identification, and validation of new targets. The overall accuracy of percutaneous biopsy is 90–95%; higher positive recovery rates govern biopsy of osteolytic lesions. The rate of complications for percutaneous biopsy approaches is <5%. The purpose of this review is to provide information about performing bone biopsy and what to expect from it as well as choosing the appropriate imaging guidance. Additionally, factors governing the appropriate needle trajectory that would likely give the greatest diagnostic yield and choice of the most appropriate biopsy system and type of anesthesia will be addressed.

## 1. Introduction

Bone metastasis is the end result of a cascade of events including tumor cell seeding and dormancy as well as metastatic growth; the bone marrow microenvironment can act as a reservoir for malignant cells [1]. Metastatic bone disease is most commonly seen with cancer arising from the breast, prostate, lung, and kidney, as well as multiple myeloma; the most common sites of bone metastases are throughout the axial skeleton [1,2].

Percutaneous, imaging-guided biopsy of bone metastasis is a minimally invasive diagnostic technique that can be proposed, among others, for characterization and identification of a suspicious lesion. In the spine, percutaneous postero-lateral blind biopsy approaches have been performed since the mid-1930s by Robertson and Ball; fluoroscopically- and computed tomography-guided biopsies were introduced in everyday clinical practice since 1949 and 1981, respectively [3]. Nowadays, there is an increasing demand for biopsies mainly due to the need for confident histological diagnosis and due to the possibility of cancer changing its biological behavior [4]. Open biopsies run the risk of destabilizing an already diseased spinal or peripheral skeleton segment. Percutaneous biopsies obviate such issues and provide immediate confirmation of correct needle location in the area of interest.

The purpose of this review is to provide information about performing bone biopsies and what is to be expected from them as well as choosing the appropriate imaging guidance. Pubmed central database was searched using the terms ‘bone’, ‘metastases’, and ‘biopsy’. Additionally, factors governing the appropriate needle trajectory that would likely give the greatest diagnostic yield and choice of the most appropriate biopsy system and type of anesthesia will be addressed.

## 2. Indications—Contraindications

Indications for percutaneous biopsy of a bone lesion include, but are not limited to, assessment of benign versus malignant bone or vertebral lesion/fracture, determining the nature of a lesion with intermediate or aggressive imaging features, confirmation of metastatic tumor involvement of a bone in a patient with a known primary neoplasm, confirmation of multiple myeloma diagnosis [5,6,7,8]. Specifically for breast carcinoma, metastatic disease percutaneous biopsy can be also applied to confirm concordance (or discordances) of the disease’s biological features and tumor characteristics [9]. Additional indications in the era of personalized medicine include identification of new targets, optimal treatment, and tumor recurrence as well as prediction of tumor response and recurrence rate. Predicting recurrence in curative cases could help in treatment stratification, identification and validation of new targets. Absolute contraindications are rare and include lack of a safe access, uncorrected coagulopathy, and patient refusal to consent [6].

## 3. Pre-Procedural Imaging

Pre-procedural imaging should include at least one cross sectional and/or functional study which depending on the clinical issue should include computed tomography (CT), magnetic resonance imaging (MRI), or positron emission tomography CT (PET/CT). Evaluation of pre-procedural imaging will provide information governing selection of optimal imaging guidance method, assessment of an appropriate needle trajectory that would likely give the greatest diagnostic yield and selection of most appropriate biopsy system and type of anesthesia. Additional issues to be addressed by pre-procedural imaging evaluation include the patient’s position, scheduled number of samples, identification of potential contraindications, and risks and anticipation of possible complications [6].

## 4. Techniques

**Figure 1:**
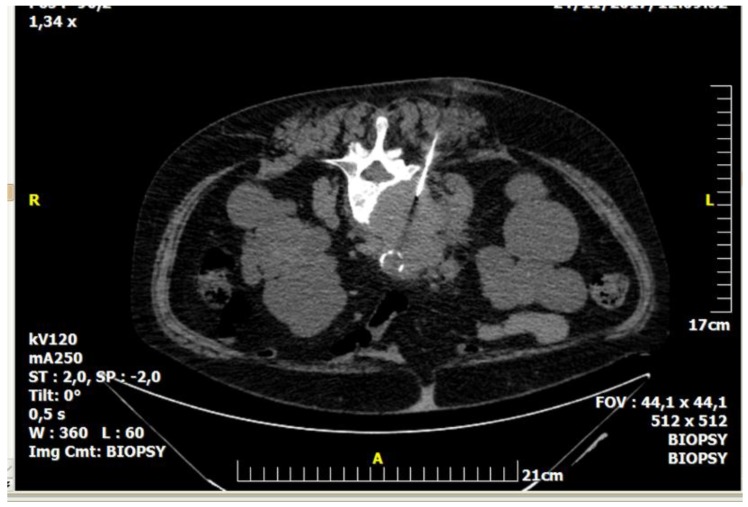
A 59-year-old female patient with a medical record of urothelial carcinoma. Computed tomography axial scan: there is a soft tissue mass infiltrating the L4 vertebral body. The result of percutaneous, imaging-guided biopsy was metastasis from urothelial carcinoma.

**Figure 2:**
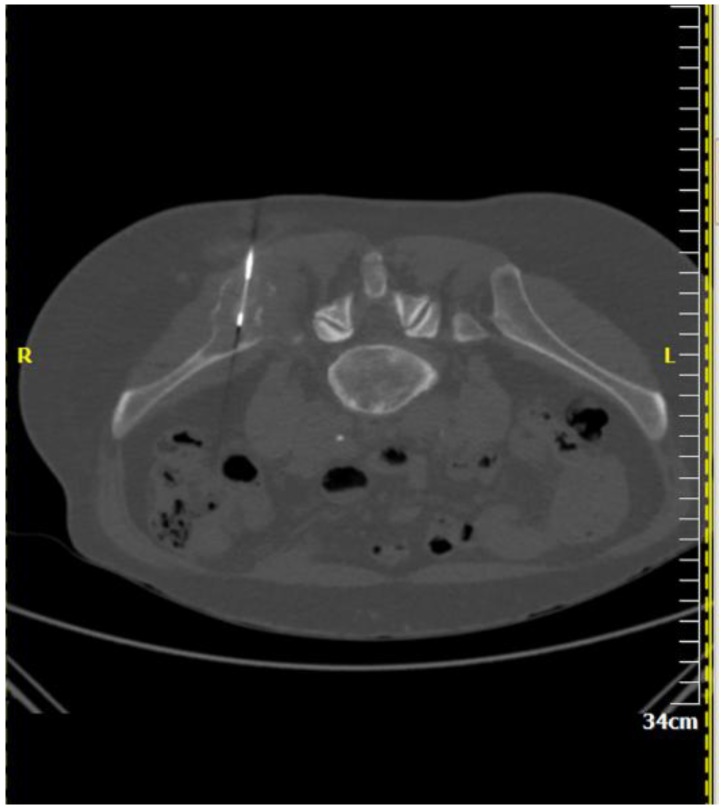
A 64-year-old male patient with multiple osteolytic lesions. Computed tomography axial scan: there is a lytic lesion in the right iliac bone. The result of percutaneous, imaging-guided biopsy was metastasis from small cell bronchogenic carcinoma.

A detailed description of percutaneous biopsy technique is beyond the scope of this review. However, due to their importance, certain technical factors should be addressed (Table 1). Percutaneous biopsy of bone lesion should be performed with the patient in a comfortable position (choice of which depends on the selected access route) and under local sterility measures. Choice of imaging guidance method depends on the lesion’s size, location, and characteristics as well as upon availability and operator’s preferences; imaging methods to be used include ultrasound, fluoroscopy, CT or MRI, flat-panel cone beam CT, as well as fusion imaging (and needle tracking) or multimodality imaging on terms of any combination of the aforementioned techniques [5,6,10,11,12,13,14,15,16]. The advantages of ultrasound include lack of radiation exposure, real-time image acquisition, and widespread availability; on the other hand, image quality depends upon the operator’s skills as well as the patient’s body type. Computed tomography is also widely available, providing rapid image acquisition and improved visualization of the needle; a disadvantage is the patient’s exposure to radiation. With magnetic resonance imaging, biopsy needles can be visualized in sagittal and coronal planes with no radiation exposure although the disadvantages include a smaller working area, longer duration of the procedure, and limited availability. Types of anesthesia to be applied during percutaneous bone biopsy include local and cutaneous anesthesia, epidural anesthesia, sedation, and general anesthesia. During local anesthesia a 22-gauge spinal needle can be used to apply local anesthetic all the way to the lesion or to the bone periosteum. General anesthesia should be reserved for specific patient groups (e.g., children), very long and very painful complex cases, or cases demanding excellent respiratory control [5,6,17]. Biopsy techniques include co-axial technique or tandem approaches for fine needle aspiration or core needle biopsy sampling [6]. The choice of appropriate bone biopsy system depends mainly upon the presence of an intact cortical bone; in such a case, a trocar should be used for the coaxial approach which will also allow the operator to get multiple samples with one puncture (Figure 1 and Figure 2) [18]. Cases of a cortical bone with increased thickness and excessive periosteal reaction drilling rather than hammering could be technically easier [19,20,21].

## 5. Efficacy and Safety

Diagnostic accuracy of percutaneous bone biopsy ranges from 70–96% with a suggested threshold of 70–75% used for internal auditing [5,6,22,23,24]. Lytic character and large size of the lesion along with multiple and long specimens are important factors positively affecting the diagnostic yield [25,26]. For sclerotic lesions, drilling over manual hammering seems to increase diagnostic accuracy and yield [21]. Concerning molecular screening, imaging modality, choice of organ, and multiple samples seem to statistically affect the diagnostic yield [27].

Complications can be classified according the CIRSE (Cardiovascular and Interventional Radiological Society of Europe) classification system which grades the adverse effects on the basis of the outcome, the effect upon hospitalization duration, and severity of a specific sequel in patient’s everyday life [28]. The potential complication rate for percutaneous bone biopsy is <5% with a suggested threshold of 2% [5,6,8]. Patients’ age and gender along with lesion location are important factors governing complications rate [29]. The procedure-related mortality rate is lower than 0.05% [5,6]. A list of complications post bone biopsy includes but is not limited to bleeding, infection, surrounding organ perforation, and tract seeding. Specifically for tract seeding, tumor type and location as well as needle size and the number of needle passes are important factors affecting the potential rate [5].

## 6. Conclusions

The majority of all cancer patients will eventually present bone metastases; spine is the most common site of osseous metastatic disease. Percutaneous approaches include minimally invasive techniques which obviate the risk of destabilizing an already diseased spinal or peripheral skeleton segment; furthermore, imaging guidance will provide immediate confirmation of correct needle location in the area of interest. Nowadays, percutaneous biopsy of bone metastases apart from being an established procedure with high efficacy and safety rates additionally has an evolving role for personalized cancer care.

## Figures and Tables

**Table 1:** Technical factors concerning percutaneous biopsy of bone metastases.

**Imaging Guidance**	Fluoroscopy (incl. Cone beam CT)	Ultrasound (incl. Fusion imaging)	Computed Tomography (incl. CT fluoroscopy)	Magnetic Resonance Imaging
**Biopsy Techniques**	Co-axial technique	Tandem technique	Fine needle aspiration biopsy	Core needle biopsy
**Diagnostic Accuracy**	70–96% depending upon target’s size and location, benign or malignant character, number of samples, on-site presence of cytopathologist			
**Complications**	Procedure related mortality rate <0.05%—quality improvement threshold for overall incidence of complication of 2%			
**References**	Veltri et al. CVIR 2017: CIRSE guidelines on percutaneous needle biopsy [6]		Gupta et al. JVIR 2010: Quality improvement guidelines for percutaneous needle biopsy [5]	
